# Correlational study on novelty factor, immersive tendencies, purchase intention and memory in immersive VR e-commerce applications

**DOI:** 10.1038/s41598-023-36557-8

**Published:** 2023-07-14

**Authors:** Guilherme Gonçalves, Galvão Meirinhos, Miguel Melo, Maximino Bessa

**Affiliations:** 1 Institute for systems and computer engineering technology and science, HUMANISE, Porto, 4200-465 Portugal; 2 Department of Engineering, University of Trás-os-Montes e Alto Douro, Vila Real, 5000-801 Portugal

**Keywords:** Human behaviour, Information technology

## Abstract

E-commerce is a field that changed how consumers purchase and interact with products. Although, inherent limitations such as the difficulty of testing the products “first-hand” before a purchase can compromise consumers’ trust in online purchases. Virtual Reality (VR) has been investigated as a tool to solve limitations in several fields and how we can harness its potential to improve the overall user experience. This study analysed how immersive VR (IVR) could solve these limitations by allowing consumers to test products beforehand. We have studied how the Novelty Factor (evaluated by the users’ past VR experience) and Immersive Tendencies correlate with the users’ Purchase Intention and Memory (how well they remember the product’s characteristics). We have analysed a sample of 38 participants (21 males) from 18 to 28 years old. Participants experienced a refrigerator with an interactive touchscreen in an IVR setup and were guided through its functionalities. Results indicated that memory of the product’s characteristics was positively correlated with how recently they experienced VR. No correlations were found in the female sample. A negative correlation between Purchase Intention and Memory of the product’s characteristics was found in the male sample. We concluded that IVR applications could become helpful for both consumers and online shops in an e-commerce context regardless of the Novelty Factor and Immersive Tendencies of consumers. However, differences between genders should be further investigated.

## Introduction

Virtual Reality (VR) potential has been explored in several fields (e.g. rescuers^1^, medical^2^, educational^3^, marketing^4^) due to its capabilities to create virtual experiences where users behave similarly to real analogous situations. VR, more specifically, immersive VR (IVR), VR using immersive technologies such as head-mounted displays (HMD), can isolate users entirely from real-world stimuli^5–7^. Therefore, users experimenting with IVR can lose awareness of their surroundings and become focused and immersed in the virtual world as if it was their “new reality”. Technology has evolved so that society went from expensive and bulky VR equipment^8^ to accessible high-quality HMDs with great support from the community and developers worldwide. Developing VR applications is becoming easier and more profitable as research keeps increasing on VR and authoring tools^9,10^ together with increasing adoption of VR^11^. Lightweight IVR applications can even be run in smartphones equipped with adaptors such as a VR cardboard viewer and in standalone solutions such as Oculus Quest (recently named Meta Quest). Using such devices increases mobility and reduces the logistics behind setting up desktop computer-based HMD at the potential cost of quality.

E-commerce drastically changed how consumers purchase products. There are multiple marketplaces where consumers can search for all products and investigate their characteristics and reviews from other buyers while getting products delivered to their homes. However, online shopping for physical products has the disadvantage that users can only try products firsthand when they arrive after the purchase is made. Such a disadvantage can negatively influence the consumers’ trust in buying certain types of products online, either by being expensive or because the online description is not enough for the consumer to make a fully informed purchase. Kim et al.^12^ concluded that online shoppers with a higher degree of product-level uncertainty are more unlikely to buy expensive products online, whether or not they have previous experience purchasing online. Users are also more hesitant to purchase expensive versus cheaper products based only on the digital information available. Another study by Liu et al.^13^ has shown that buyers of luxury goods in physical stores are more hesitant about the risks of online shopping. They also consider it important to be able to check products personally before the purchase and that interactions and shopping experience are necessary.

The characteristics of IVR can help to mitigate these and other disadvantages of online shopping and also physical shopping because (a) IVR adds a safety layer with product testing being done virtually, from controlled locations, without damaging the product or harming consumers^14,15^ (e.g. testing a new chainsaw for the first time). Moreover, (b) IVR can reduce time and logistic constraints as users can experience products anywhere without travelling to the physical store. IVR is also not vulnerable to whether physical stores have available products on display, and if yes, whether they are in conditions to be tested (e.g. the functionality of a new coffee machine cannot be tested because the product on display is internally damaged). Furthermore, (c) IVR allows for collaboration in asynchronous or in real-time, where consumers can be assisted by real store staff regarding a product, where both parties can be inside the same virtual experience and see and interact between each other and the product. Lastly, (d) the “hands-on” experience in the IVR can also lead users to have more realistic expectations about the products and increase their trust and possibly reduce the number of return items^16–19^.

### Novelty factor

Although the IVR user base is increasing^20^, consumers still have never tried IVR before^21^. Using such a technology for the first time can lead to the known novelty factor (also known as the “wow factor”). Multiple studies indicated that the novelty factor could influence the user experience in multiple ways. Peixoto et al.^22^, in their systematic review of VR in foreign language education, recognised that several works have a habituation phase to address the novelty factor so that they could better focus on the task at hand afterwards. Pinto et al.^23^ work investigated the effectiveness of VR in education by comparing it to conventional learning methods (audio listening exercises) in a foreign language learning scenario. The authors concluded that results could have been influenced by whether or not it was the first time using VR for some students, which might have led to some distractions.

However, under certain circumstances, pre-exposing users are not possible or desirable, nor is the possibility only to select people that have enough previous experiences with IVR to mitigate the novelty factor. For example, online shopping would be unenforceable only to allow (or disallow) individuals with previous IVR experience to check products in IVR or make them go through a pre-exposure scene first. Therefore, it must be assumed that users at home can experiment with products using IVR whether they have enough past VR experience or not to be significantly influenced by Novelty Factor. Although, the probability of VR setup owners having enough VR experience is likely very high (as it is unlikely to purchase such equipment and not use it). However, hypothetically, family and friends without VR experience could still use those VR setups to try a product before purchasing, making this situation relevant. Furthermore, this IVR application could still be used in a physical store (either to try a product still not in stock or soon to be released) where consumers with and without experience with this technology could experience it.

For this study, Novelty Factor is considered as the extent to which the experience is considered new and different from what they have experienced before. Thus, we evaluate Novelty Factor based on how recently users have experienced IVR, their level of satisfaction with previous IVR experience and how often they play video games.

It becomes essential to analyse if purchase intention correlates with the novelty factor in IVR. This information can help e-commerce platforms to better predict purchase intentions based on how novel the use of IVR is for their customers. Consequently, different strategies can be adopted depending on the level of novelty. Another crucial factor related to the novelty factor is whether it correlates with how consumers remember their virtual experience with the product. This is due to the possible novelty factor effect on the users’ attention and focus^22,23^, which could lead to consumers becoming distracted and not paying enough attention to the product details. Consequently, a relationship between how well users remember the product details and their purchase intention should also be addressed.

With this knowledge, an e-commerce store could offer better guidance to product details and guarantee that users are fully aware of the product’s characteristics.

### Immersive tendencies

Immersive Tendencies define the propensity of subjects to experience presence^24^ (commonly known as the sense of “being there”^6^), becoming more involved in the virtual experience, and ultimately maintaining the focus on the current activities^25^. Therefore, individuals with higher Immersive Tendencies lose track of time and are more unaware of their actual surroundings. This behaviour is similar to the one predicted by the flow theory^26^. Flow is a mental state that describes subjects so deeply involved and focused on their tasks that they ignore external distractions and are unaware of the passage of time. This implies that Immersive Tendencies could influence how users process the information of the virtual environment through an increased focus on what they are doing while being less prone to outside distractions. Following this reasoning, Immersive Tendencies should influence the extent users to remember details of what they are doing by being less distracted by the complexity of the surrounding environment. For example, Mania et al.^27^ concluded that rendering quality (flat-shaded vs radiosity) influenced memory and states of awareness, with participants performing worse in the more complex visual environment (radiosity). The same reasoning is supported by Wedel et al.^28^, which argues that abstract representations might be preferable to higher realism due to the decreased visual complexity. An individual’s Immersive Tendencies can also be influenced by their life experiences, such as playing video games^24^. Therefore, their past experiences with non-immersive and IVR can correlate to how users are easily immersed in virtual experiences.

The literature has already addressed the impact of Immersive Tendencies in some contexts. For example, Krassmann et al.^29^ investigated how Immersive Tendencies could be a predictor of learning gains using a learning IVR game. Results showed there was no connection between Immersive Tendencies and learning. Similarly, Khashe et al.^30^ in their study comparing an immersive vs non-immersive virtual environment on compliance with pro-environmental behaviours, also concluded that Immersive Tendencies did not influence behaviour and performance. However, there is no literature specifically about the influence of the individuals’ Immersive Tendencies on purchase intention in an IVR setup.

Thus, it becomes crucial to analyse whether there are any correlations between Immersive Tendencies and Purchase Intention to predict purchase intentions based on an individual’s immersive tendencies and consequently adapt to the virtual experience. Because Immersive Tendencies could affect how well users become distracted from outside stimuli (and also virtual stimuli outside the task), the relationship between Immersive Tendencies with the extent users remember the details of the product should also be addressed. As discussed in the Novelty Factor subsection, further analysis of the correlation between the memory of the product details and purchase intention should also be addressed. The findings would offer valuable insights for e-commerce platforms. For example, they would provide information on whether to design immersive virtual experiences (IVEs) that better isolate users from the real world. These IVEs could enhance users’ immersion and increase their purchase intentions by enabling more informed decisions.

### Gender

Gender is still a poorly studied variable in the field of e-commerce^31^, and still, a recurring variable in VR studies^30,32–36^.

The Selectivity Hypothesis explains how both genders process information differently^37^: males and females have different thresholds regarding how much information they need to acquire before purchasing. Men tend to have a higher elaboration threshold than women, meaning that males do not need to analyse all the information about a product before purchasing it, contrary to women^37^.

Wolin et al.^38^ conducted a survey asking 420 respondents to compare web advertising to radio, newspaper, magazine, and television advertising regarding the following dimensions (enjoyable, offensive, informative, deceptive, annoying, and valuable). The authors concluded that males exhibit a more positive attitude toward online shopping than females, implying that males are more likely to purchase online.

Pascual-Miguel et al.^39^ integrate the perceived risk and trust variables into the extended unified theory of acceptance and use of technology (UTAUT2)^40^ and tested with a sample of 817 subjects in an online survey. Authors argued that male and female online shoppers appear to have a higher appreciation of online shopping and a higher perceived control when purchasing non-digital products vs digital ones. They state that the gender gap is narrowing. Nevertheless, attention should still be given to factors that impact both genders differently.

### Hypothesis

This work is part of a larger study that included other other hypotheses testing^41,42^. In Meirinhos et al.^41^ we addressed whether contextualisation of products in IVR and gender would impact purchase intention and user satisfaction; verify if purchase intention was correlated with the presence and user satisfaction; and verify if there was a correlation between user satisfaction and presence). On Gonçalves et al.^42^ we considered two independent variables (Contextualization and Gender) and in a comparative study analysed weather they had an impact on the extent users are clarified about the product functionalities and size as well as if there was an effect on the extent users remember details of the product and on the users mental workload. This paper focuses on other hypotheses, specifically verifying correlations between purchase intention and immersive tendencies and past experiences with IVR experiences (novelty factor). We also analyse whether the extent users remember the product details is correlated with immersive tendencies, novelty factors and purchase intention.

Gender was also considered as a variable. It is still poorly studied in this field^31^, and even though the differences seem to be narrowing^39^, differences between genders under IVR are still being researched. Thus, this study will verify if the correlations (or lack of) between variables will be present in both genders individually.

Therefore, we put forward the following hypothesis:

- H1—Immersive Tendencies are correlated with Purchase Intention.
- H2—Novelty Factor is correlated with Purchase Intention.
- H3—Immersive Tendencies are correlated with the extent users remember the details of the product.
- H4—Novelty Factor is correlated with the extent users remember the details of the product.
- H5—Purchase Intention is correlated with the extent users remember the details of the product.
- H6—Correlations will differ between genders.

## Methodology

The methodology is in conformity with the INESC TEC (Institute for Systems and Computer Engineering Technology and Science) ethics code checklist and FCT (Portuguese national funding agency), which provides an ethics self-evaluation guide for identifying the main ethics issues concerning research activities. Participation was voluntary and data gather was anonymized. Participants filled an informed consent form before the experiments and were told they could withdraw from the study at any point without any consequences.

### Sample

A non-probabilistic convenience sampling method was used to recruit 38 volunteers (21 males) aged between 18 and 28 years ($M=21.370, S.D.=2.541$). Most subjects were university students ($86.8\%$), with the rest being workers. The sample was gathered at the University of Trás-os-Montes e Alto Douro between March and April 2022. The product being tested in this study is a refrigerator; thus, we asked participants whether they had ever purchased this type of product before themselves. The majority confirmed that they did not (73.7%). All participants finished the study successfully, as there were no withdrawals.

### Variables

The variables considered in this study can be checked in table 1. The variables included in the correlation study are Purchase Intention, Novelty Factor (Last IVR Experience, Satisfaction with IVR Experiences, Video Game Playing Frequency), Memory (Correct Answers and Level of Confidence), and Immersive Tendencies (Focus, Game, Involvement, and overall Immersive Tendencies score).

**Table 1:** Description variables in the study and their sub-scales.

Variables	Description	Sub-scales
Novelty Factor	The past VR experience of the subjects and how prone they could be to the novelty factor	When was the last VR experience, satisfaction with their previous VR experiences, how often subjects play video games
Immersive Tendencies	Tendencies of individuals to experience presence^24^	Focus, Game, Involvement, and Overall
Purchase intention	The extent users are willing to purchase the product	None
Memory	The extent users were aware of details about their experience with the product.	Correct answers and level of confidence in the answers
Gender	Biological gender of participants.	None

### Instruments

Novelty Factor is based on the user’s past VR experience, more specifically, when was the last time they experienced VR (with the answers: Never, more than 1 year ago, between 6 months to 1 year ago, between 1 year and 6 months ago, less than 1 month ago), what was their level of satisfaction with the experience rated in a Likert scale of 5-points, how often they play video-games (never, every month, every week, every day up to 1 h, every day more than 1 h). Satisfaction with IVR experiences was only evaluated if the participant had a previous experience with VR. This information was evaluated through a sociodemographic questionnaire. The same questionnaire also addressed whether subjects have ever purchased a refrigerator and other sociodemographic information such as gender, age, and occupation.

To evaluate Immersive Tendencies, we applied the translated version of the Immersive Tendencies Questionnaire (ITQ) by Witmer & Singer^24^. It comprises 18 questions on a 7-point Likert scale with the following subscales (focus, game, involvement, and overall immersive tendency score).

Purchase Intention was evaluated using one question, based on Spangenberg et al.^43^ work, rated on a 7-point Likert scale from very unlikely to very likely: “If you were going to purchase a refrigerator/this type of product, how likely would you be to purchase this particular product”.

Memory evaluation questions were based on a study by Mania et al.^27^ with the following multiple-choice (four choices) questions in the questionnaire: “What was the brand of the refrigerator?”, “How many years of warranty had?”, “How many drawers had?”, “How many compartments did the freezer have?” and “What was the lowest possible temperature for the freezer?”. For each question, users also reported their confidence in their answer using a 5-point Likert scale ranging from “No confidence” to “Certain”. The Correct Answers subscale score was calculated by summing all correct answers (between 0 and 5 points, 1 point per correct answer). The level of Confidence was calculated by averaging the level of confidence for each question (min of 1 and max of 5 per question, where 5 is the max level).

### Materials

**Figure 1:**
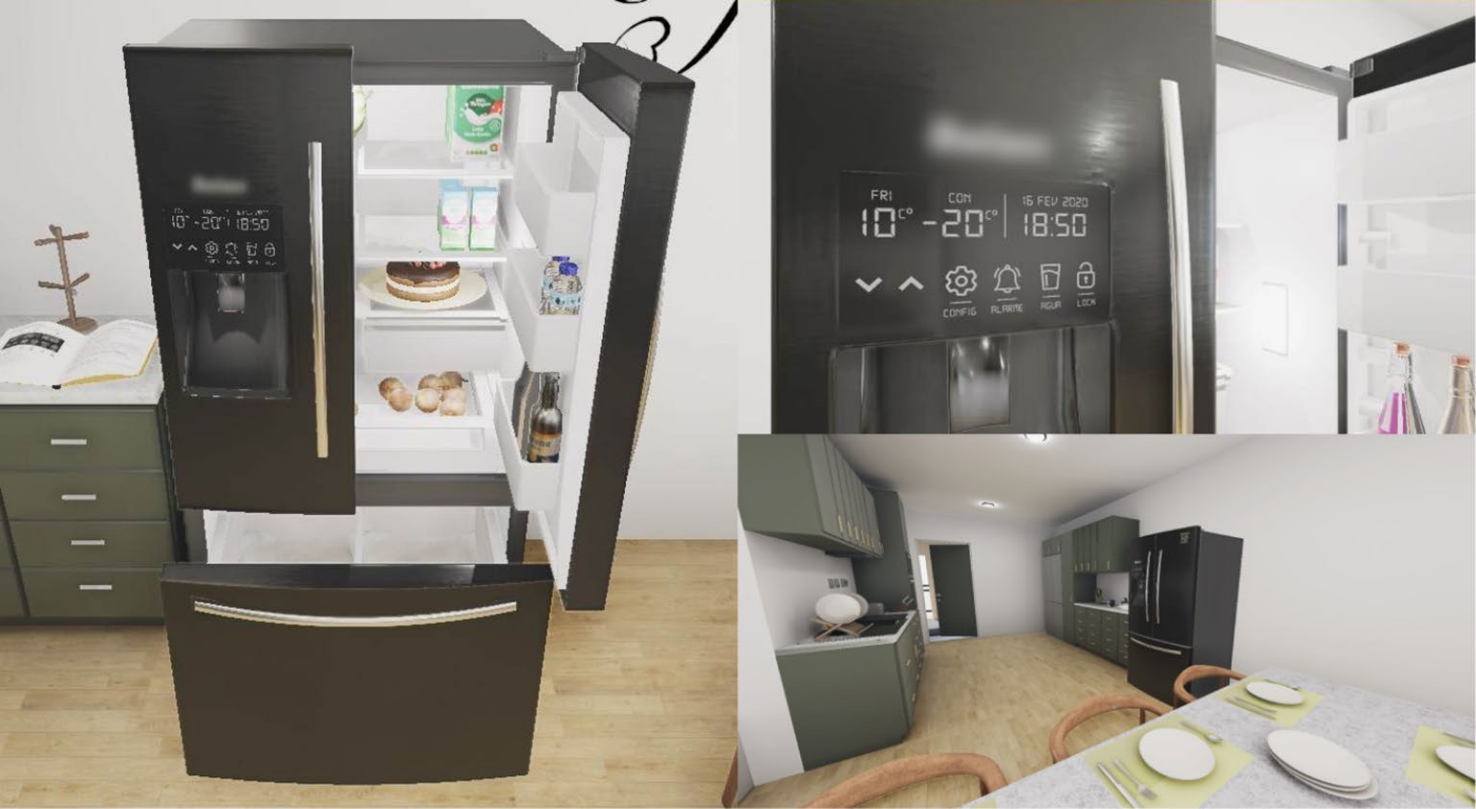
Left: exterior and interior of the refrigerator. Right top: interactive touchscreen. Right bottom: contextual virtual environment surrounding the refrigerator.

The product and virtual environment were the same as the ones in Meirinhos et al.^41^ and Gonçalves et al.^42^. The product was a double-door refrigerator with a touchscreen, ice/water dispenser, and freezer. The touchscreen allowed the participants to check the set temperature for the refrigerator and freezer, the current hour and date, set up an open door alarm, change the dispenser mode between ice and water, and lock/unlock the touchscreen. There were two possible environments: a neutral one, where the refrigerator was presented empty in a clean white room, and a contextual one (Fig. 1), where the same refrigerator had food products inside and was presented in a room with kitchen appliances and furniture. The refrigerator in the contextual virtual environment and its touchscreen can be visualized in Fig. 1. The VR application was developed on Unity 2022 using the high-definition render pipeline.

The VR headset was an HTC Vive Pro (1440 $\times$ 1600@90hz per eye), equipped with a wireless module and the respective controllers. The computer had an Intel Core i7-8700K processor, 32 GB RAM, and an NVIDIA GeForce RTX 2080ti, using Windows 10.

**Table 2:** Description of every step participants performed during the product analysis.

Step	Description
1	Check the exterior of the refrigerator
2	Open the refrigerator doors and check inside
3	Open the refrigerator drawers, check the inside, and close them again
4	Close the refrigerator doors
5	Open the freezer drawer, check inside, and close it again
6	Pick up the refrigerator touchscreen manual
7	With the help of the manual, perform the following tasks
7.1	Decrease the refrigerator temperature to 3 $^{\circ }$C
7.2	Decrease the freezer temperature to the minimum allowed
7.3	Update the hour and date to the current one (given by the researcher)
7.4	Set the open door alarm to 1 m 30 s
7.5	Deactivate the open door alarm
7.6	Lock and unlock and touchscreen
7.7	Change the dispenser mode to ice
8	Grab a cup near the refrigerator and dispense some ice into it

### Procedures

Participants were debriefed about what they would do without disclosing the purpose and variables of the study. They then started by filling out a consent form, sociodemographic questionnaire, and ITQ. Participants were then explained how to use the controllers, and after they acknowledged they understood, they were equipped with the VR headset. The VR application was executed, and participants were transported into the virtual experience facing the refrigerator. Due to the methodology used to gather data for the hypotheses testing on Meirinhos et al.^41^ and Gonçalves et al.^42^, half of the participants experimented with the product in a neutral context and the other half in a kitchen context, balanced by gender. Instructions to participants were given verbally in the order found in Table 2.

The experiment ended after all tasks were completed. There was no time limit, and participants were told to take as long as needed. After being unequipped with the help of a researcher, participants filled out the purchase intention and memory questionnaires.

### Informed consent

Informed consent was obtain from every participant.

## Results

The statistical procedure was performed using JASP 0.16.2 open-source statistics program. Data from the Immersive Tendencies was missing for one participant, being an exclude cases pairwise used in the correlation analysis (the participant was not included in correlations between Immersive Tendencies and other variables). Spearman’s correlation was performed to verify correlations between variables. The monotonic relationship assumption was verified through a visual inspection of scatterplots. Initially, we considered the totality of the sample for the correlations (H1–H6), and then we performed the same correlation tests in the male and female samples separately (H7). Descriptive statistics (Means and Standard Deviations) are shown in Table 3. Correlation results are displayed in Table 4, displaying Spearman’s coefficient, significance level, and sample count for the total, male, and female samples.

**Table 3:** Descriptive Statistics.

Variables	Subscales	Full sample		Males		Females	
		Mean	S.D.	Mean	S.D.	Mean	S.D.
Novelty factor	Freq. games	2.053	1.610	3.000	1.225	0.882	1.219
	Past VR Exp.	1.316	1.544	1.857	1.621	0.647	1.169
	Past VR Exp. satisfaction	3.227	0.685	3.125	0.719	3.500	0.548
Immersive tendencies	ITQ focus	18.947	4.926	20.762	2.625	16.706	6.152
	ITQ games	8.632	4.857	12.000	2.739	4.471	3.448
	ITQ involvement	18.026	5.222	19.905	2.844	15.706	6.527
	ITQ immersive tendencies	63.132	15.654	70.333	7.172	54.235	18.713
Purchase intention		5.526	1.133	5.333	0.913	5.765	1.348
Memory	Correct answers	3.421	1.030	3.571	0.926	3.235	1.147
	Confidence	3.895	0.618	3.943	0.559	3.835	0.697

**Table 4:** Spearman’s correlation coefficient and significance for each variable pair between full sample, male sample, and female sample.

Pair	Full sample			Males			Females		
	$r_s$	*p*	*n*	$r_s$	*p*	*n*	$r_s$	*p*	*n*
H1									
Purchase intention $\times$ focus	− 0.099	0.560	37	0.370	0.099	21	− 0.191	0.480	16
Purchase intention $\times$ games	− 0.295	0.076	37	− 0.079	0.735	21	− 0.325	0.219	16
Purchase intention $\times$ involvement	0.114	0.501	37	0.151	0.512	21	0.109	0.687	16
Purchase intention $\times$ immersive tendencies	− 0.090	0.598	37	0.299	0.187	21	0.009	0.973	16
H2									
Purchase intention $\times$ last VR exp	− 0.181	0.276	38	− 0.185	0.421	21	− 0.100	0.702	17
Purchase intention $\times$ VR satisfaction	− 0.203	0.365	22	− 0.301	0.257	16	0.104	0.845	6
Purchase intention $\times$ video-game frequency	− 0.240	0.147	38	− 0.190	0.410	21	0.125	0.631	17
H3									
Focus $\times$ correct answers	− 0.074	0.664	37	− 0.349	0.121	21	− 0.145	0.592	16
Focus $\times$ confidence	0.060	0.724	37	0.023	0.921	21	− 0.130	0.632	16
Games $\times$ correct answers	0.215	0.202	37	0.259	0.257	21	− 0.036	0.896	16
Games $\times$ confidence	0.155	0.361	37	0.122	0.599	21	− 0.176	0.514	16
Involvement $\times$ correct answers	− 0.003	0.987	37	− 0.183	0.428	21	0.134	0.621	16
Involvement $\times$ confidence	0.233	0.165	37	0.208	0.366	21	0.293	0.271	16
Immersive tendencies $\times$ correct answers	− 0.025	0.884	37	− 0.295	0.194	21	− 0.103	0.703	16
Immersive tendencies $\times$ confidence	0.141	0.404	37	0.155	0.504	21	-0.005	0.985	16
H4									
Video-game frequency $\times$ correct answers	0.226	0.173	38	0.167	0.470	21	0.233	0.367	17
Video-game frequency $\times$ confidence	0.071	0.674	38	− 0.103	0.657	21	0.091	0.727	17
Last VR exp $\times$ correct answers	**0.353**	**0.030**	38	**0.461**	**0.035**	21	0.244	0.345	17
Last VR exp $\times$ confidence	0.136	0.414	38	0.109	0.639	21	0.037	0.887	17
VR satisfaction $\times$ correct answers	0.007	0.975	22	0.141	0.602	16	− 0.422	0.405	6
VR satisfaction $\times$ confidence	0.173	0.441	22	0.222	0.408	16	0.098	0.854	6
H5									
Purchase intention $\times$ correct answers	− 0.100	0.550	38	$-$ **0.581**	**0.006**	21	0.402	0.110	17
Purchase intention $\times$ confidence	0.069	0.679	38	− 0.224	0.330	21	0.350	0.168	17

Significant values are in [bold].

## Discussion

Discussion is divided into six subsections, each one dedicated to one hypothesis.

### H1—Immersive tendencies $\times$ purchase intention

In H1, we expected that the user’s Immersive Tendency would be correlated with Purchase Intention. The more users feel immersed, the more they focus on what they are doing in the virtual environment (in this case, analysing a product) and become less prone to distractions from the real physical world. Such could affect their purchase intention. The results showed that none of the Immersive Intentions subscales was found to be correlated with Purchase Intention, rejecting H1. It could be that the simplicity of the tasks users performed did not require substantial focus; therefore, their completion would not benefit from users being immersed and focused. Experiments were also conducted in a controlled laboratory room, built specifically to isolate users from the real world and avoid outside distractions pulling users away from the virtual experience. This fact might have reduced the importance of a higher immersive tendency to overcome distractions.

Another reason could be that users knowing the product better by staying focused on it does not guarantee that they would like it better. In other words, knowing a product in-depth could warrant that users dislike it even more. For example, one could like a specific product at first sight, but when trying it out, become aware that it was not as great as previously thought. The same can happen otherwise. Whichever the case, becoming more informed about the product would be the objective of such an IVR application because: (a) it could decrease the number of online purchases being returned due to dissatisfaction and (b) possibly improve the users’ trust towards buying products they would otherwise be hesitant to buy before trying. Either way, these results suggest that the consumer’s Immersive Tendencies do not predict their Purchase Intention when shopping using IVR setups.

### H2–Novelty factor $\times$ purchase intention

We also hypothesized in H2 that Novelty Factor would be correlated with Purchase Intention. This was due to the “wow factor” users experience when trying IVR without being accustomed to it. Such could result in users being positively surprised and excited, which could raise expectations about the product they are testing and wanting to buy through this medium because it is perceived as innovative. No correlations were found between the variables that compose the Novelty Factor and Purchase Intention; therefore, H2 is rejected. In this case, the novelty was on the medium to purchase a product, not the product itself. So although users can be intrigued by the novelty and want to try it out, it can also happen that users just prefer the familiar way to purchase products since they are more comfortable with it.

Such a result suggests that users with less experience with IVR should be no different from experienced users regarding their Purchase Intentions. If the case, there would be no need to pre-expose users to IVR environments to reduce the Novelty Factor and control their expectations. However, we should note that there are still several reasons some users should be pre-exposed to IVR in e-commerce applications (for example, providing a simple virtual environment where they can learn to use the controllers and interact without the distractions of the surrounding virtual environment). Whether users choose to be pre-exposed or not, this provides evidence that it should not influence their Purchase Intention either way. Also, based on the study results, it may be less effective to target specific audiences less experienced with IVR to increase purchase intention. Further research is still needed better to understand the relationship between Novelty Factor and Purchase Intention.

### H3—Immersive tendencies $\times$ memory

Users with higher Immersive Tendencies tend to be more focused on the tasks; therefore, we hypothesised that this increased focus and consequent loss of awareness of the surroundings of the virtual and real environment would allow users to acknowledge more details about the product they are analysing (details related and unrelated with the tasks) and at the end of the experience recall them better (H3). Results indicated no correlation between Immersive Tendencies subscales and Correct Answers scores and Confidence scores, thus rejecting H3. Such indicates that the extent one becomes more easily immersed in IVEs should not affect their level of attention towards the details of the product. There is also the possibility that participants might not have wanted this product, which would result in a lack of interest in the details. Another possible reason could be that the product is standard equipment, which might have grabbed less attention. More studies are needed to verify these results; however, if such is the case, it would benefit clients and sellers as it would be one less variable to affect the purchase experience in an IVE setup. For example, subjects less attentive to product details due to their immersive tendencies could have a higher chance of purchasing the product with the wrong notion of its characteristics, thus increasing the chance of product returns and implying costs to buyers and sellers.

### H4—Novelty factor $\times$ memory

In H4, we hypothesized that Novelty Factor would influence how well participants remember the details of their experience with the product. Literature suggests that the Novelty Factor can be one of the factors that led users to have lower learning scores than expected. This is because the participants wander around in the virtual environment and are less focused on the essential information being conveyed to them^22,23^. Therefore we expected that Novelty Factor would be related to the users’ performance in recalling the product’s characteristics correctly and confidently. The results led us to accept H4 partially, as there was a positive correlation between how recently they had their previous VR experience and the level of correct answers. It also seems to partially corroborate the statements of Peixoto et al.^22^ and Pinto et al.^23^ in that participants seem to recall better the essential information the IVR environment is aimed to provide the less the Novelty Factor impact (measured by how recently the participants had their previous VR experience). Home users of IVR setups will likely have a vast experience with IVR and experience it often enough not to be influenced by the Novelty Factor. Therefore, users trying products this way will have a better chance of acknowledging product characteristics and details and, thus, becoming better informed before any purchase decision. However, it also suggests that there should be caution when consumers with little VR experience try products virtually (such as in a physical store or by trying setups from other establishments or friends/family), as they could create a wrong perception of the product. In this situation, proper guidance towards the product’s characteristics should be present, ensuring the correct information is acquired. Nevertheless, further studies are required to verify if the same holds accurate with more complex products that may require more attention to detail as well as whether the type of VR experience that users last had may impact their ability to acquire and retain product information.

### H5—Purchase intention $\times$ memory

The results did not support the hypothesis that purchase intention is correlated with product knowledge retention and confidence in recall (H5). One possible explanation is that participants may not have been motivated to purchase as they knew the VR experience was a simulation for a scientific study or because they were not actively shopping for a refrigerator. This lack of motivation may have resulted in less attention paid to product details and less confidence in the recall. While having a correct perception of a product is important, it does not necessarily mean consumers will like it more. In fact, it is possible that a correct perception could lead to a dislike of the product, which could cancel out any correlation with Purchase Intention. Regardless, the VR application would still serve its purpose of providing consumers with product information when physical testing is not possible or convenient. In addition, future studies could examine other types of products more appealing to the target audience (varying in price and complexity) to gain further insights.

### H6—Correlations for each gender

Gender is a recurring variable in e-commerce and advertising. Studies suggest that genders differ in how they perceive and process information before purchasing. Therefore, this work also addressed gender and analysed whether correlations differ between male and female samples (H6).

In the male sample, there was a significant negative correlation between the time of the last VR experience and the number of correct answers on the recall test, indicating that the more recent the last VR experience was, the higher the score. This relationship was expected in both genders. The selectivity hypothesis^37^ could have been the underlying cause for no correlations in the female sample; however, further studies are needed to verify this result. It could be possible that because females tend to analyse more in-depth information about products than men, the novelty factor might have influenced them less. In contrast, in the male sample, this novelty factor could have had a higher impact on their ability to focus on product characteristics.

Lastly, Purchase Intention was found to be correlated (negatively) with Correct Answers only in the male sample. The lower their score in the recall test about the product characteristics, the less their purchase intention. We cannot make a causal relation as their purchase intention could have influenced how well they were focused on the product characteristics in the first place, or the fact that they did not acknowledge all the characteristics of the product could have led to a lower purchase intention (which could be unlikely as knowing the product better could also mean consumers dislike it more). The female sample did not present this correlation, and one of the possible justifications could be once again related to the selectivity hypothesis^37^. In Meirinhos et al.^41^, we already concluded that Purchase Intention was not different between genders. Therefore, we can assume that females having a higher overall purchase intention for the product and thus being more focused on the product characteristics than males could not have been the reason for them not to be influenced by this correlation. Further investigation is needed to understand these gender differences and whether the selectivity hypothesis could have affected the results.

## Conclusions and future work

This study aimed at understanding how Novelty Factor (evaluated by the past VR and video game experience of users) and Immersive Tendencies are correlated with Purchase Intention and Memory (how well users recall the characteristics of the product) in an IVR setup showcasing a product in the context of an e-commerce application. Purchase Intention was also investigated to determine whether it was correlated with Memory. We also analysed if these correlations were present in both genders.

We concluded that neither Immersive Tendencies nor Novelty Factors were correlated with Purchase Intention (H1 and H2). This means that consumers’ Immersive Tendencies data should not predict how well they are prone to purchase products presented in an IVR scenario. Furthermore, familiarity with the technology (IVR) also seems to not affect users’ purchase intention, suggesting that efforts to target consumers without as much experience with IVR to incentivise purchases could be less relevant.

We found no evidence that the Correct Answers and Confidence scores in the recall test are correlated with Immersive Tendencies (H3). However, the time that passed since the participants had their previous VR experience was found to be correlated with how well participants remembered the details of the product (Correct Answers scores in the recall test) (H4). This suggests that the more recent the last experience with IVR, the higher their recall performance (likely due to the decreased novelty factor impact). It also suggests that guidance should be provided to users more prone to the Novelty Factor, ensuring they acknowledge the product characteristics correctly.

No correlations were found between Purchase Intention and Memory. Further studies are required to understand this result better and verify whether the extent users acknowledge the product characteristics pulled them away or not from their intent to purchase. The analysis should include other products, including more complex products and products that users appreciate. Nevertheless, the main objective of such a VR application would still hold: provide enough information for an informed decision.

Only the male gender displayed significant correlations, specifically in the relation between Novelty Factor and Memory and between Purchase Intention and Memory. This suggests that genders differ in some key aspects that could become relevant in using IVR for e-commerce. Further studies should be considered to understand these differences better, specifically if the selectivity hypothesis could have had an effect.

Ultimately, the main goal of such an application could still be reached: provide a tool for users to become better informed about products they cannot test physically before purchasing.

## Limitations

This work had limitations that should be addressed in future iterations. The sample used was university students between 18 and 28 years old, an age group known to adhere to new technologies. The behaviours of participants who were interested in the showcased product could differ from those who were not. Participants were also aware that this was an experiment and no product could be purchased, which could have changed how they processed information regarding the product they were testing. Furthermore, the product had no price or other information that could be relevant (such as power consumption). The IVR application focused instead on the product’s functionality as a tool to give consumers a notion of its size and functions. The significant correlations found can also be due to other variables’ influence and not specifically between the pair of variables being studied. The sample size, when split by gender, was low, which may have affected the results. The variable VR Satisfaction only had data from participants that had previous experience with VR. Because the female sample had less experience with VR, the sample count used for pairs including this variable was even lower.

## Data Availability

The datasets used and/or analysed during the current study available from the corresponding author on reasonable request.

## References

[1] Narciso D, Melo M, Raposo JV, Cunha J, Bessa M (2020). Virtual reality in training: An experimental study with firefighters. Multimedia Tools Appl..

[2] Aïm F, Lonjon G, Hannouche D, Nizard R (2016). Effectiveness of virtual reality training in orthopaedic surgery. Arthroscopy J. Arthrosc. Relat. Surg..

[3] Freina, L. & Ott, M. A literature review on immersive virtual reality in education: State of the art and perspectives. In *The International Scientific Conference eLearning and Software for Education, vol. 1* 133 (“ Carol I” National Defence University, 2015).

[4] Kim TH, Choo HJ (2021). Augmented reality as a product presentation tool: Focusing on the role of product information and presence in ar. Fashion Textil..

[5] Milgram P, Kishino F (1994). Taxonomy of mixed reality visual displays. IEICE Trans. Inf. Syst..

[6] Slater M (2009). Place illusion and plausibility can lead to realistic behaviour in immersive virtual environments. Philos. Trans. R. Soc. B: Biol. Sci..

[7] Gonçalves G, Coelho H, Monteiro P, Melo M, Bessa M (2022). Systematic review of comparative studies of the impact of realism in immersive virtual experiences. ACM Comput. Surv..

[8] Heilig Morton, L. *Sensorama Simulator* (United States), https://lens.org/101-000-708-415-191 (1962).

[9] Coelho H, Monteiro P, Gonçalves G, Melo M, Bessa M (2022). Authoring tools for virtual reality experiences: A systematic review. Multimedia Tools Appl..

[10] Goncalves G, Monteiro P, Coelho H, Melo M, Bessa M (2021). Systematic review on realism research methodologies on immersive virtual, augmented and mixed realities. IEEE Access.

[11] Alsop, T. *[*Virtual Reality (vr)—Statistics & Facts, https://www.statista.com/topics/2532/virtual-reality-vr/#topicOverview (Statista, 2022).

[12] Kim Y, Krishnan R (2015). On product-level uncertainty and online purchase behavior: An empirical analysis. Manage. Sci..

[13] Liu X, Burns AC, Hou Y (2013). Comparing online and in-store shopping behavior towards luxury goods. Int. J. Retail Distrib. Manage..

[14] Monteiro P, Melo M, Valente A, Vasconcelos-Raposo J, Bessa M (2020). Delivering critical stimuli for decision making in vr training: Evaluation study of a firefighter training scenario. IEEE Trans. Hum.-Mach. Syst..

[15] Narciso, D., Melo, M., Rodrigues, S., Cunha, J. P. S. & Bessa, M. Impact of different stimuli on user stress during a virtual firefighting training exercise. In *2020 IEEE 20th International Conference on Bioinformatics and Bioengineering (BIBE)* 813–818 (IEEE, 2020).

[16] Shulman JD, Cunha M, Saint Clair JK (2015). Consumer uncertainty and purchase decision reversals: Theory and evidence. Mark. Sci..

[17] Foscht T, Ernstreiter K, Maloles C, Sinha I, Swoboda B (2013). Retaining or returning? some insights for a better understanding of return behaviour. Int. J. Retail Distrib. Manage..

[18] Rao S, Rabinovich E, Raju D (2014). The role of physical distribution services as determinants of product returns in internet retailing. J. Oper. Manag..

[19] Suh K-S, Lee YE (2005). The effects of virtual reality on consumer learning: An empirical investigation. Mis Q..

[20] Heaney, D. *Steam Users with A vr Headset Passes 2% for First Time*, https://uploadvr.com/steam-vr-users-2-percent/ (UploadVR, 2021).

[21] Navaratnam-Blair F, Wagstaff K, Miller G, Cumberbatch M, Rethore C (2022). Beyond reality is the long-awaited vr revolution finally on the horizon?.

[22] Peixoto B, Pinto R, Melo M, Cabral L, Bessa M (2021). Immersive virtual reality for foreign language education: A prisma systematic review. IEEE Access.

[23] Pinto, D. *et al.* Virtual reality in education: Learning a foreign language. In *World Conference on Information Systems and Technologies* 589–597 (Springer, 2019).

[24] Witmer BG, Singer MJ (1998). Measuring presence in virtual environments: A presence questionnaire. Presence.

[25] Banerjee P, Bochenek GM, Ragusa JM (2002). Analyzing the relationship of presence and immersive tendencies on the conceptual design review process. J. Comput. Inf. Sci. Eng..

[26] Csikszentmihalyi, M. *Flow: The Psychology of Optimal Experience, vol. 1990* (Harper & Row, 1990).

[27] Mania K, Wooldridge D, Coxon M, Robinson A (2006). The effect of visual and interaction fidelity on spatial cognition in immersive virtual environments. IEEE Trans. Vis. Comput. Graph..

[28] Wedel M, Bigné E, Zhang J (2020). Virtual and augmented reality: Advancing research in consumer marketing. Int. J. Res. Mark..

[29] Loureiro Krassmann, A. *et al.* Learning in virtual reality: Investigating the effects of immersive tendencies and sense of presence. In *International Conference on Human-Computer Interaction* 270–286 (Springer, 2020).

[30] Khashe, S., Becerik-Gerber, B., Lucas, G. & Gratch, J. Persuasive effects of immersion in virtual environments for measuring pro-environmental behaviors. In *ISARC. Proceedings of the International Symposium on Automation and Robotics in Construction, vol. 35* 1–7 (IAARC Publications, 2018).

[31] Lin X, Featherman M, Brooks SL, Hajli N (2019). Exploring gender differences in online consumer purchase decision making: An online product presentation perspective. Inf. Syst. Front..

[32] Melo, M., Gonçalves, G., Narciso, D. & Bessa, M. Impact of different role types and gender on presence and cybersickness in immersive virtual reality setups. In *2021 International Conference on Graphics and Interaction (ICGI)* 1–8 (IEEE, 2021).

[33] Coelho, H., Melo, M., Branco, F., Vasconcelos-Raposo, J. & Bessa, M. The Impact of gender, avatar and height in distance perception in virtual environments. In *New Knowledge in Information Systems and Technologies, vol. 931. Series Title: Advances in Intelligent Systems and Computing* 696–705. 10.1007/978-3-030-16184-2_66 (Springer International Publishing, Cham, 2019).

[34] Melo M, Vasconcelos-Raposo J, Bessa M (2018). Presence and cybersickness in immersive content: Effects of content type, exposure time and gender. Comput. Graph..

[35] Felnhofer A, Kothgassner OD, Beutl L, Hlavacs H, Kryspin-Exner I (2012). Is virtual reality made for men only? exploring gender differences in the sense of presence. Proc. Int. Soc. Presence Res..

[36] Gonçalves, G., Melo, M. & Bessa, M. Virtual reality games: A study about the level of interaction vs. narrative and the gender in presence and cybersickness. In *2018 International Conference on Graphics and Interaction (ICGI)* 1–8 (IEEE, 2018).

[37] Meyers-Levy J, Maheswaran D (1991). Exploring differences in males’ and females’ processing strategies. J. Consum. Res..

[38] Wolin LD, Korgaonkar P (2003). Web advertising: Gender differences in beliefs, attitudes and behavior. Internet Res..

[39] Pascual-Miguel FJ, Agudo-Peregrina ÁF, Chaparro-Peláez J (2015). Influences of gender and product type on online purchasing. J. Bus. Res..

[40] Venkatesh V, Thong JY, Xu X (2012). Consumer acceptance and use of information technology: Extending the unified theory of acceptance and use of technology. MIS Q..

[41] Meirinhos G, Gonçalves G, Melo M, Bessa M (2022). Using virtual reality to demonstrate and promote products: The effect of gender, product contextualization and presence on purchase intention and user satisfaction. IEEE Access.

[42] Gonçalves G, Meirinhos G, Filipe V, Melo M, Bessa M (2022). Virtual reality e-commerce: Contextualization and gender impact on user memory and user perception of functionalities and size of products. IEEE Access.

[43] Spangenberg ER, Crowley AE, Henderson PW (1996). Improving the store environment: Do olfactory cues affect evaluations and behaviors?. J. Mark..

